# Therapeutic Assay with the Non-toxic C-Terminal Fragment of Tetanus Toxin (TTC) in Transgenic Murine Models of Prion Disease

**DOI:** 10.1007/s12035-021-02489-5

**Published:** 2021-07-20

**Authors:** Marina Betancor, Laura Moreno-Martínez, Óscar López-Pérez, Alicia Otero, Adelaida Hernaiz, Tomás Barrio, Juan José Badiola, Rosario Osta, Rosa Bolea, Inmaculada Martín-Burriel

**Affiliations:** 1 Centro de Encefalopatías Y Enfermedades Transmisibles Emergentes, Universidad de Zaragoza, IA2, IIS Aragón, 50013 Zaragoza, Spain; 2 Laboratory of Genetics and Biochemistry (LAGENBIO), Faculty of Veterinary, Institute for Health Research Aragon (IIS Aragón), AgriFood Institute of Aragon (IA2), University of Zaragoza, Miguel Servet 177, 50013 Zaragoza, Spain; 3 Centro de Investigación Biomédica en Red de Enfermedades Neurodegenerativas (CIBERNED), Instituto Carlos III, Madrid, Spain; 4 Instituto de Investigación Biomédica de Bellvitge (IDIBELL), L’Hospitalet de Llobregat, Barcelona, Spain; 5 UMR Institut National de La Recherche Pour L’Agriculture, L’Alimentation Et L’Environment (INRAE)/École Nationale Vétérinaire de Toulouse (ENVT) 1225 IHAP (Interactions Hôtes-Agents Pathogènes), 31076 Toulouse, France

**Keywords:** Prion, Prion diseases, Neurodegeneration, Autophagy, Tetanus toxin

## Abstract

The non-toxic C-terminal fragment of the tetanus toxin (TTC) has been described as a neuroprotective molecule since it binds to Trk receptors and activates Trk-dependent signaling, activating neuronal survival pathways and inhibiting apoptosis. Previous in vivo studies have demonstrated the ability of this molecule to increase mice survival, inhibit apoptosis and regulate autophagy in murine models of neurodegenerative diseases such as amyotrophic lateral sclerosis and spinal muscular atrophy. Prion diseases are fatal neurodegenerative disorders in which the main pathogenic event is the conversion of the cellular prion protein (PrP^C^) into an abnormal and misfolded isoform known as PrP^Sc^. These diseases share different pathological features with other neurodegenerative diseases, such as amyotrophic lateral sclerosis, Parkinson’s disease or Alzheimer’s disease. Hitherto, there are no effective therapies to treat prion diseases. Here, we present a pilot study to test the therapeutic potential of TTC to treat prion diseases. C57BL6 wild-type mice and the transgenic mice Tg338, which overexpress PrP^C^, were intracerebrally inoculated with scrapie prions and then subjected to a treatment consisting of repeated intramuscular injections of TTC. Our results indicate that TTC displays neuroprotective effects in the murine models of prion disease reducing apoptosis, regulating autophagy and therefore increasing neuronal survival, although TTC did not increase survival time in these models.

## Introduction

Transmissible spongiform encephalopathies (TSEs), also termed prion diseases, are fatal neurodegenerative disorders caused by prions that affect humans and a wide variety of animal species [1]. These diseases share, as the main pathogenic event, the conversion of the cellular prion protein (PrP^C^) into an abnormal and misfolded isoform known as PrP^Sc^. This leads to the accumulation of PrP^Sc^ in the central nervous system (CNS), which causes neuronal dysfunction and cell death [2]. TSEs show morphological and pathophysiological hallmarks that mirror those of other neurodegenerative diseases, such as amyotrophic lateral sclerosis (ALS), Alzheimer’s disease or Parkinson’s disease [3].

Neurodegeneration is the main pathological feature of prion diseases. Although molecular mechanisms involved in the pathogenesis of prion diseases are still unknown, several pathways have been proposed to play an important role in TSE-associated neurodegeneration, among which autophagy and apoptosis are particularly relevant [4].

Autophagy is an essential mechanism of cellular catabolism in which damaged components, such as misfolded proteins, are included in double-membrane structures, known as autophagosomes, and degraded by lysosomes hence maintaining homeostatic balance inside cells [5]. Thus, the activation of autophagic mechanisms seems to play a protective role since it can efficiently degrade misfolded proteins [6], and many studies have shown its importance in different neurodegenerative disorders like amyotrophic lateral sclerosis, Alzheimer’s disease or Parkinson’s disease, in which the presence of abnormal protein aggregates is a common feature [7]. In prion diseases, it has been observed that the impairment of autophagic mechanisms pharmacologically or by siRNA inhibits the capacity of cells to degrade PrP^Sc^ [6]. Many studies have identified autophagic dysregulation in TSE models [4, 8–11], including the model used in this study [12] and impaired protein homeostasis has been implicated as an important major cause of toxicity common to prion diseases [13]. Autophagic mechanisms, originated as a survival response to intracellular PrP^Sc^ accumulation, can fail to recover homeostasis after exposure to PrP^Sc^, thus leading to neuronal dysfunction and cell death [14].

The activation of pro-apoptotic pathways has been demonstrated in natural forms of prion disease [15]. These mechanisms have proven to be a common cause of neuronal cell death in animal TSEs in different studies of scrapie-infected sheep, mice and hamsters [16, 17]. They have also been reported as a common form of neuronal death in human prion diseases as Creutzfeldt-Jakob disease (CJD) and fatal familial insomnia (FFI) [18]. Although its role is still controversial, apoptosis in prion diseases has been linked to the up-regulation of the pro-apoptotic factor BAX (BCL2 associated X) [19, 20]. Apoptosis can also be induced by chronic endoplasmic reticulum stress, which could activate pro-apoptotic pathways, such as IRE1 (inositol-requiring enzyme 1), ATF6 (activating transcription factor 6) or Akt/PI3K (protein kinase B/phosphatidylinositol 3-kinase). All of them are related to the chaperone BiP/GRP78 (binding immunoglobulin protein), reported to be overexpressed in this kind of diseases [21]. Moreover, prion protein has been directly linked to apoptosis since PrP^Sc^ aggregates have been associated with activation of caspase 3 and caspase 8 proteins, triggering caspase-dependent pathways and resulting in apoptosis [22].

Tetanus neurotoxin (TeNT), a protein produced by *Clostridium tetani*, is the causative agent of tetanus, a potentially fatal condition [23]. TeNT toxicity depends on the presence of an L chain with metalloprotease activity [24], but its targeting to the neuronal synapses relies only on a 50 kDa carboxy-terminal non-toxic fragment known as TTC which is not only required but sufficient for neuron binding and internalization [25], retrograde transport and transsynaptic transport [26]. Hence, TTC has been used for targeting therapeutic molecules to the CNS [27], and several molecules such as the insulin-like growth factor 1 (IGF-1), the glial-derived neurotrophic factor (GDNF) or the brain-derived neurotrophic factor (BDNF), among others, have been successfully transported into neurons by linking them to TTC [28–30].

Moreover, further studies have shown that TTC is more than a carrier, and it also has neurotrophic properties. TTC mimics the action of natural neurotrophic ligands by binding to Trk receptors and activating Trk-dependent signaling, which leads to the activation of neuronal survival pathways and apoptosis inhibition [31]. Even though the detailed mechanism on how TTC activates Trk receptors is not known, activation of neuroprotective pathways by TTC binding to these receptors has been demonstrated in both in vivo and in vitro studies [32, 33]. TTC alone delays the onset of symptoms and functional deficits in ALS murine models, decreasing motoneuron death and prolonging mice lifespan [30, 34] and in mouse models of spinal muscular atrophy (SMA), where it modulates autophagy markers and reduces apoptosis [35].

The aim of the present study was to test TTC as a potential therapy for prion diseases. Considering the impact of autophagy and apoptosis in both prion diseases [36] and ALS [34, 37], in which TTC has shown therapeutic results, and the potential of TTC as an anti-apoptotic and autophagy-regulator molecule, we assessed the apoptotic and autophagic mechanisms in the CNS of prion diseased murine models treated with TTC.

## Materials and Methods

### Murine clinical Assay

Two models of mice were used: C57BL6 (expressing the wild-type variant of murine PrP^c^) and Tg338 transgenic mice (overexpressing ~ 8 × the VRQ variant of ovine PrP^C^) [38]. Twenty-four animals of the Tg338 mouse line were intracerebrally inoculated with a second passage of sheep scrapie in Tg338, while twelve C57BL6 mice were intracerebrally challenged with the scrapie strain 22L.

Each mouse line was separated into two different groups: a control group (Tg338 *n* = 12; C57BL6 *n* = 6) and a treated group (Tg338 *n* = 12; C57BL6 *n* = 6). The treated group was intramuscularly inoculated with 1 µg of TTC recombinant protein [39] in the quadriceps muscle once a month, for 6 months, while the control group was intramuscularly inoculated with PBS following the same pattern. Groups were sex-balanced, with approximately half males and half females in all of them. Following the experimental design performed in previously published assays with TTC in neurodegenerative diseases [34, 39], no negative controls (prion uninfected mice, treated and untreated) were included since no neurodegeneration was supposed to appear at the ages the animals were sacrificed, and therefore no TTC-related effects were expected.

Mice were monitored daily for the onset of neurological signs. All animals were humanely euthanized by cervical dislocation when clinical signs of disease were detected (i.e. locomotor disorders, poor body condition and any signs of impaired feeding ability), and their brains collected. Brains were sagittally divided into two, and half of the brain was preserved in formaldehyde for its further use in histopathological and immunohistochemical analyses, while the other half was divided in two parts, one containing the frontal cortex, which was preserved frozen for further biomolecular analyses, and the other containing the rest of the areas, which was collected in RNAlater™ Solution for its use in gene expression analyses. No significant signs of distress or pain were observed in the animals other than those related to the onset of neurological signs, at which time they were euthanized. Kaplan–Meier survival curves were drawn and statistically significant differences in survival curves were assessed with the Mantel–Cox log-rank test (*p* < 0.05).

### Histological and Immunohistochemical Analyses

The presence and distribution of PrP^Sc^ in mouse brain samples was evaluated by paraffin-embedded tissue-blot (PET-blot). PET-blot was performed as described elsewhere [40]. Briefly, 4-μm paraffin-embedded brain sections were collected onto a nitrocellulose membrane and dried at 56 °C for 24 h. Membranes were then subjected to dewaxing and rehydration and incubated for 2 h in a proteinase K solution (250 μg/ml) at 56 °C to completely digest PrP^C^. Denaturation of the remaining PrP^res^ was achieved by incubating the membranes in a solution of guanidine thiocyanate 3 M. After blocking the membrane with 0.2% BSA to avoid cross-reactivity, detection was carried out through sequential incubation with the anti-PrP antibody Sha31 (1:8,000, SPI-Bio) and a secondary alkaline phosphatase-conjugated antibody (1:500, Dako Agilent, Santa Clara, CA, USA), followed by development with NBT/BCIP (Thermo Fisher Scientific, Waltham, MA, USA).

To perform immunohistochemical and histopathological studies, sagittal sections from paraffin-embedded mouse brains (4 μm thick) were cut and collected on glass slides and dried at 56 °C for 24 h. Immunohistochemistry was used for the detection of NeuN, the activated (cleaved) form of caspase-3, LC3B and p62 to assess neuronal survival, apoptosis, and autophagic mechanisms, respectively. Antigen retrieval was performed with citrate buffer (pH 6.0) for 10 min at 96 °C, and endogenous peroxidase activity was blocked using a blocking reagent (Dako Agilent) for 15 min. Next, sections were incubated with primary antibodies anti-LC3B (1:200; Santa Cruz Biotechnology, Dallas, TX, USA, sc-271625), anti-p62 (1:200; Enzo Life Sciences, PW9860) and anti-NeuN (1:100, Abcam, Cambridge, UK, ab104224) during 1 h at room temperature and anti-caspase-3 (1:50; Santa Cruz Biotechnology, sc-56052) overnight at 4 °C. Then, samples were incubated with an enzyme-conjugated Envision polymer (Dako Agilent) for 30 min at room temperature and DAB (diaminobenzidine, Dako Agilent) was used as the chromogen. For each marker, all animals were stained in a single batch in order to reduce possible staining intensity differences due to the immunohistochemistry performance. The specificity of the immunohistochemical technique was determined by the absence of immunostaining in mouse brain sections in which the primary antibody incubation was omitted.

Neuropathological changes and spongiform lesions were studied by hematoxylin–eosin staining.

Brain sections were examined using a Zeiss Axioskop 40 optical microscope. Evaluation was blindly performed in all animals, in nine encephalic areas : frontal cortex (Fc), septal area/striatum (Sa), Parietal cortex (Pc), hippocampus (Hc), thalamus (Th), hypothalamus (Ht), mesencephalon (Mes), medulla oblongata (Mobl) and cerebellum (Cbl) [41]. The cerebellum was not evaluated for LC3B, p62 and NeuN immunohistochemistry since no immunostaining was found in this area. Spongiosis and PrP^Sc^ deposition were semi-quantitatively scored on a scale of 0 (lack of spongiosis/PrP^Sc^ deposit) to 5 (very intense spongiosis/PrP^Sc^ deposit) since semiquantitative evaluation is the standard method to assess these features [42]. Semiquantitative evaluation for spongiosis and PrPSc was performed twice for each mouse and brain area. Caspase-3 staining was quantitatively evaluated by counting all the stained cells present in the whole sagittal section of each mouse brain at a 20 × magnification. LC3B, p62 and NeuN immunostaining was assessed using the Image J software. The Image J assessed markers staining was evaluated by the colour deconvolution method as previously described [43], which measures both the number of stained cells and the intensity at once. Two photographs (taken at 10 × magnification) of each brain area per mouse were evaluated. The used chromogen in the immunohistochemistry techniques was diaminobenzidine (DAB), and therefore the images were subjected to colour deconvolution using the “H DAB” algorithm, and the “Colour_2” image (showing the DAB staining) intensity colour was measured by the “Mean gray value”. Once the mean gray value for each photograph was obtained, the resulting intensity values were converted to optical density by using the following formula: OD = log(maximum intensity/mean intensity), therefore quantifying the average darkness of the image due to DAB signal. Since empty areas within the image can bias the results reducing the average OD, only photographs in which tissue occupied the whole area were analysed. Statistically significant differences were determined using the Mann–Whitney *U* test (*p* < 0.05).

### Western Blot Analyses

Briefly, 25 μg of total protein from the frontal cortex of four mice per group were subjected to 10% SDS/PAGE and transferred to PVDF membranes (Bio-Rad, Hercules, CA, USA). After blocking at 4 °C overnight with 2% bovine serum albumin (Thermo Fisher Scientific), the membranes were incubated for 1 h at RT with the following primary antibodies diluted in blocking buffer: mouse monoclonal anti MAP-LC3B (1:1000; Santa Cruz Biotechnology, sc-271625) and rabbit polyclonal anti p62 (1:1000, Enzo Life Sciences, PW9860). Next, the membranes were incubated for 1 h at RT with a HRP conjugated secondary antibody diluted 1:4000 in blocking buffer (goat anti-mouse IgGHRP for MAP-LC3, or goat anti-rabbit IgG-HRP for p62; Santa Cruz Biotechnology). After washing, Western blots were developed using the ECL Plus Western Blotting system (GE Healthcare, UK) and visualized with VersaDoc imaging system (Bio-Rad). After detection of the interest protein, β-actin immunodetection was also performed in both membranes to normalize band intensity quantification. Membranes were washed with PBST for 1 h at room temperature and then incubated with mouse monoclonal anti β-actin antibody (1:2000, Bio-Rad, VMA00048) for 2 h at room temperature and developed as described above.

The image J software was also used to quantify band intensity in Western blots. For this purpose, each band was individually selected and circumscribed with the rectangular ROI (region of interest) selection and then “Gels” function was used, obtaining the corresponding histograms for each band. This was followed by quantification of the peak area of intensity of the histograms. Obtained values were normalized through the measure of the intensity of β-actin bands. Statistically significant differences were determined using the *Student’s t* test (*p* < 0.05).

### Gene Expression Analyses

Four genes described to be involved in autophagy were selected for the analysis of their expression profile in the brain of mice: *Atg5 (autophagy related 5), Becn1 (Beclin 1), Fbxw7 (F-box/WD repeat-containing protein 7)* and *Gas5 (growth arrest-specific 5).* Gene expression was quantified in the thalamic area (including thalamus, hypothalamus and the surrounding area) of mice since it showed a high accumulation of PrP^Sc^ and high scores of spongiform changes in both mouse models.

Sagittally cut sections of the thalamic area (thalamus, hypothalamus and surrounding area) were collected in RNAlater™ Solution (Thermo Fisher Scientific). Total RNA was obtained from 80 mg of this area using an RNeasy Lipid Tissue Mini kit (QIAGEN®, Venlo, the Netherlands) and following the manufacturer’s recommended protocol. Complementary DNA (cDNA) was obtained from 200 ng of total RNA using qScript™ cDNA Supermix (Quanta Biosciences™, Beverly, MA, USA). Resulting cDNA was diluted 1:5 in water for further analyses. The quantitative real-time polymerase chain reaction (qRT-PCR) was performed with TaqMan probes (Thermo Fisher Scientific) used for the amplification of the selected genes. PCR reactions were performed using TaqMan Universal PCR Master Mix (Thermo Fisher Scientific), and PCR was carried out in a StepOne Real-Time PCR System (Thermo Fisher Scientific) using universal conditions. All reactions were run in triplicate in a total volume of 5 μL, using 2.25 μL of diluted cDNA. The expression of two housekeeping genes (*H6pd, Sdha*) was used to normalize results. Relative gene expression quantification was subsequently determined using normalized data and relative quantification was performed using the 2^−ddCt^ method. Differences between experimental groups were evaluated using an unpaired Student’s *t* test (two-tailed), with significance at *p* < 0.05.

## Results

### Survival Time, Histopathological Lesions and PrP^Sc^ Deposition Do Not Significantly Variate After Intramuscular Injection of TTC in Prion  Infected Mice

**Fig. 1:**
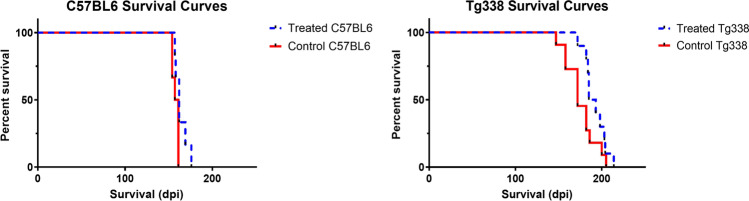
Effect of TTC injection in the survival of prion-infected mice. Figure shows Kaplan–Meier survival curves for C57BL6 mice and Tg338 mice. The Mantel–Cox log-rank test revealed no significant differences between groups

All mice were sacrificed at the onset of clinical signs. Signs of disease in C57BL6 included severe ataxia and poor body condition, while Tg338 showed slight locomotor disorders, kyphosis and poor body condition. For C57BL6 mice, the mean survival time for the treated group (*n* = 6) was 164 ± 7 days, while for the control group (*n* = 6) was 157 ± 4 days. Regarding Tg338 mice, mean survival times were 192 ± 12 days for the treated group (*n* = 12) and 176 ± 16 days for the control group (*n* = 12). Although survival time means were higher for the treated group in both Tg338 and C57BL6, no significant differences between treated and control mice survival periods were found in any of the mouse lines when applying the Mantel-Cox log-rank test (Fig. 1).

Regarding prion-associated neuropathology, all mice of each line displayed similar neuropathological lesion profiles and PrP^Sc^ deposition patterns, despite belonging to the treated or control group.

**Fig. 2:**
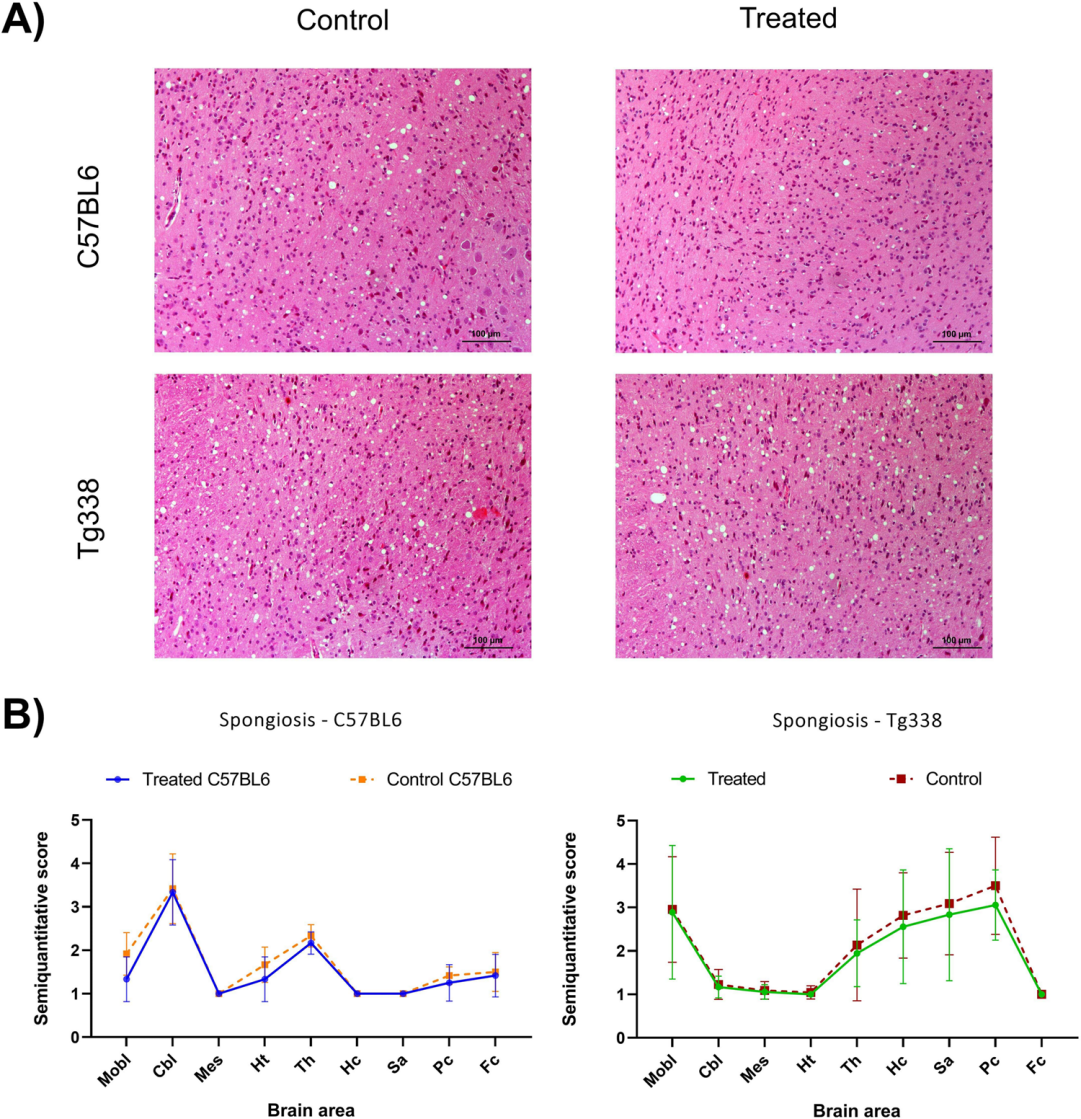
Spongiform changes in the brains of TTC-injected and control Tg338 and C57BL6 mice. **A** Representative images of hematoxylin and eosin staining in the hypothalamus of clinical prion-infected C57BL6 and Tg338 mice. **B** Graphics show comparisons of the semiquantitative evaluation (on a scale of 0, lack of spongiosis, to 5, very intense spongiosis) of these lesions in TTC-injected and control groups of both mouse lines in medulla oblongata (Mobl), cerebellum (Cbl), mesencephalon (Mes), hypothalamus (Ht), thalamus (Th), hippocampus (Hc), septal area/striatum (Sa), Parietal cortex (Pc) and frontal cortex (Fc). Spongiosis levels are similar in the control and treated groups. No significant differences were found using the Mann–Whitney *U* test

Spongiosis, the main histopathological lesion in prion diseases, was semiquantitatively evaluated in nine brain areas of all groups of mice on a scale from 0, meaning an absence of vacuolization, to 5, meaning intense spongiosis (Fig. 2A). Spongiform changes were mostly observed in the neuropil rather than in neurons. C57BL6 mice showed higher spongiform changes in cerebellum and thalamus, while in Tg338 mice the most severe lesions were observed in medulla oblongata, thalamus, hippocampus, parietal cortex and septal area/striatum (Fig. 2B). Although treated mice seemed to display slightly lower lesion degrees in some brain areas in both C57BL6 and Tg338 mouse lines, no significant differences were found between the treated and control groups in any of the lines.

**Fig. 3:**
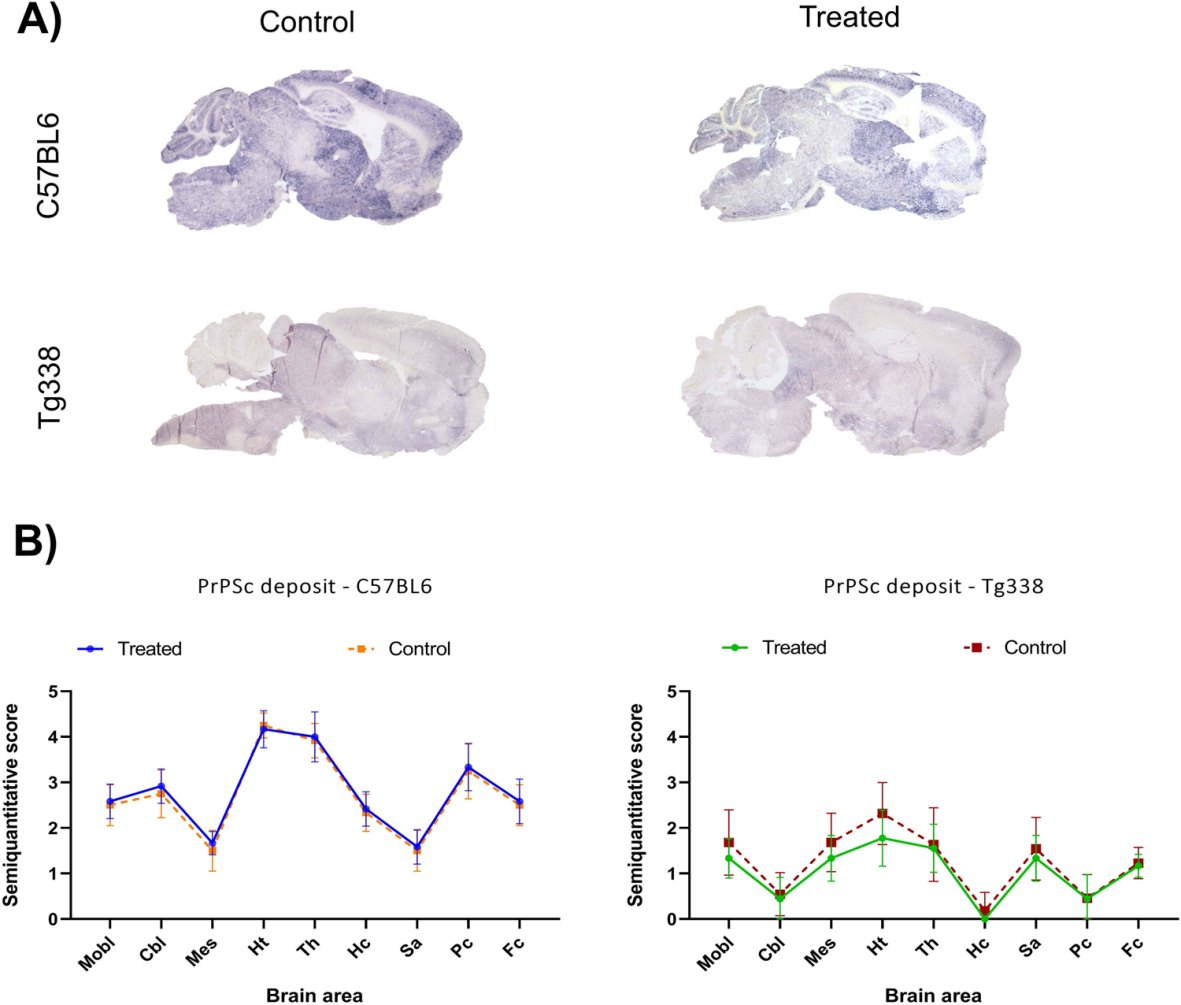
PrP^Sc^ deposition in the brains of Tg338 and C57BL6 mice TTC-injected and control groups. **A** Representative images of PrP^Sc^ deposits detected by PET-blot method in the brain of C57BL6 and Tg338 mice. **B** Graphics show comparisons of the semiquantitative evaluation (on a scale of 0, lack of staining, to 5, staining present at maximum intensity) of these deposits in TTC-injected and control groups of Tg338 and C57BL6 mice in medulla oblongata (Mobl), cerebellum (Cbl), mesencephalon (Mes), hypothalamus (Ht), thalamus (Th), hippocampus (Hc), septal area/striatum (Sa), Parietal cortex (Pc) and frontal cortex (Fc). Mice belonging to each of the lines showed similar deposition scores. No significant differences of PrP^Sc^ deposition were found using the Mann–Whitney *U* test

Concerning PrP^Sc^ deposits, evaluated by PET-Blot and semiquantitative scoring, C5BL6 mice showed higher deposition at the cerebellum, cortex, thalamus and hypothalamus, while in Tg338 mice deposition profiles were characterized by peak scores at the hypothalamus, thalamus, mesencephalon and medulla oblongata (Fig. 3A). Deposition scores were similar in control and TTC-treated mice, and no statistically significant changes were detected comparing groups of each mouse line in any of the brain areas (Fig. 3B).

### The Pro-apoptotic Activated Form of Caspase-3 Is Reduced in TTC-Treated Mice with a Clinical Prion Disease

**Fig. 4:**
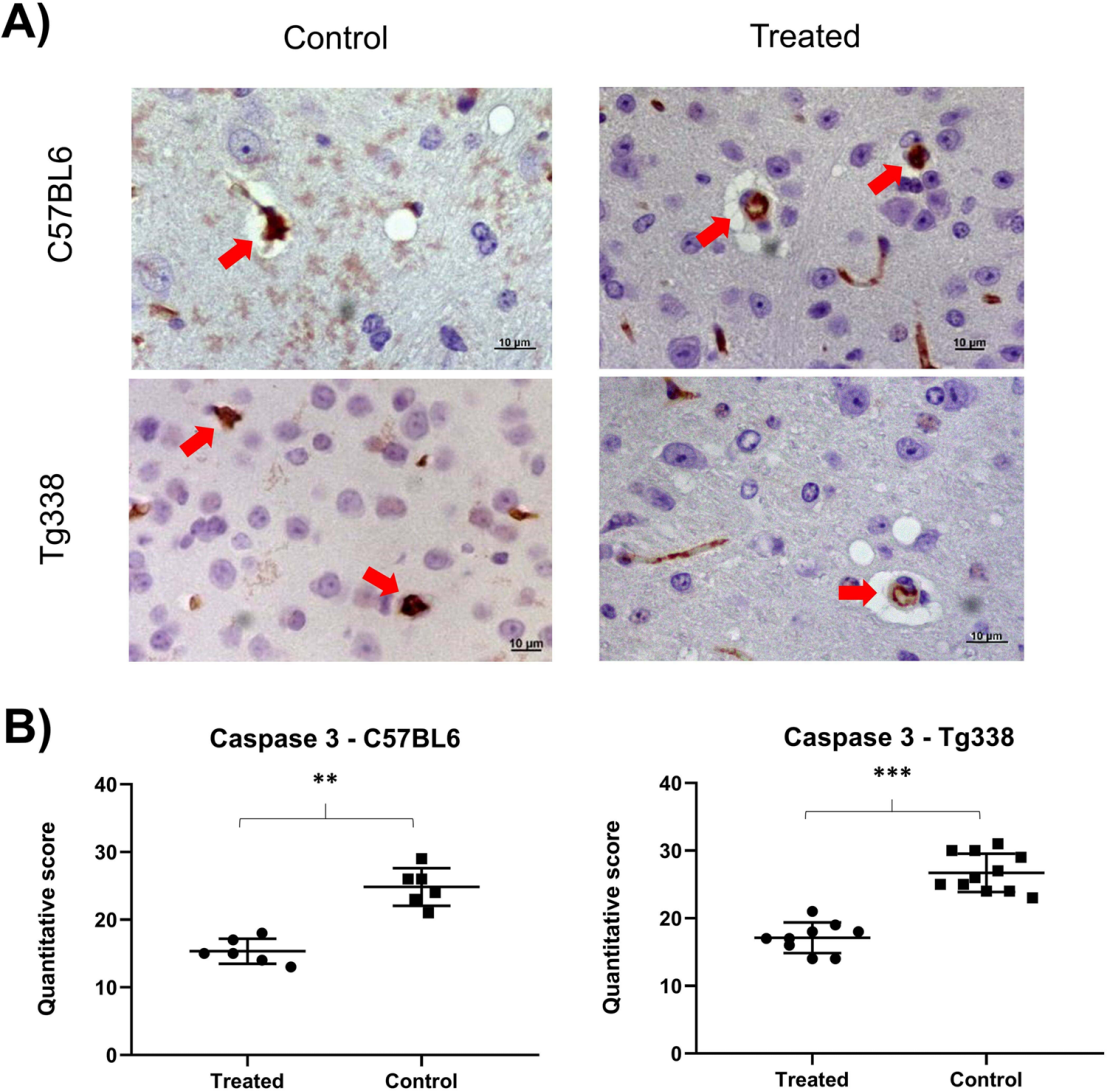
Caspase-3 expression levels in CNS are higher in control mice than in TTC-treated mice. **A** Caspase-3 immunopositive cells (arrows) in the central nervous system of mice. **B** Graphics show the quantitative determination of caspase-3 stained cells in the brains of treated and control groups of C57BL6 and Tg338 mice. Both mouse lines showed a lower number of immunopositive cells in the treated group, and significant differences appeared between groups when testing them with the Mann–Whitney *U* test (**p < 0.01 and ***p < 0.001)

Immunohistochemical assessment of the active form of caspase-3 revealed an increase of apoptotic processes in non-treated mice compared to TTC-injected animals. Caspase-3 immunostaining revealed isolated immunopositive neurons scattered throughout the brain. Apoptotic nuclei were also observed in some stained cells. Stained neurons were quantified and the treated animals, both in line C57BL6 and in line Tg338, presented a significant lower number of apoptotic cells (Fig. 4). When statistically compared with the Mann–Whitney *U* test, significant differences were found between the TTC-treated and control group in both mouse lines.

### Mice Treated with Intramuscular Injections of TTC Show Differential Regulation of Autophagy Markers in the Brain Compared to Controls

**Fig. 5:**
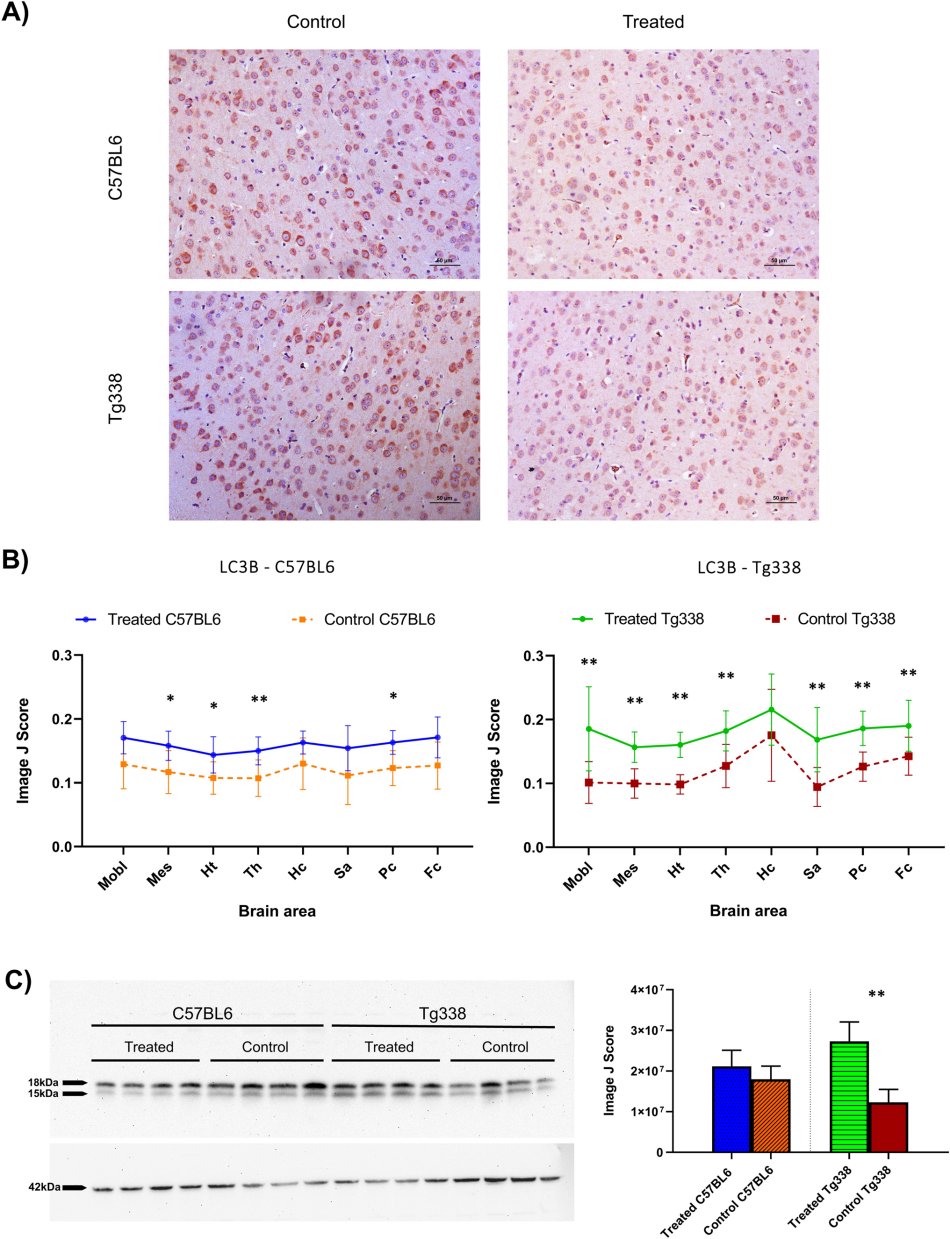
LC3B determination in Tg338 and C57BL6 prion infected mice after TTC intramuscular injection. **A** Different staining levels of LC3B immunostaining found in mice cerebral cortex, showing a cytoplasmic staining in neurons. **B** Comparison of the evaluation through Image J software of the LC3B immunostaining in TTC-injected and control groups of Tg338 and C57BL6 lines in medulla oblongata (Mobl), mesencephalon (Mes), hypothalamus (Ht), thalamus (Th), hippocampus (Hc), septal area/striatum (Sa), parietal cortex (Pc) and frontal cortex (Fc). Treated mice of both lines show higher LC3B accumulation. Significant differences were studied using the Mann–Whitney *U* test (*p < 0.05 and **p < 0.01) and found in Mes, Ht, Th and Pc in C57BL6 mice and in Mobl, Mes, Ht, Th, Pc, Sa and Fc in Tg338 mice. **C** Western Blot analyses of LC3B in the frontal cortex of the four groups of mice. Distinctive bands at ~ 18 kDa and ~ 15 kDa, corresponding to the lipidated and non-lipidated forms of LC3B. β-Actin immunodetection is shown at ~ 42 kDa. Significant differences were found between treated and untreated mice in the Tg338 line when using the Student’s *t* test (**p < 0.01)

LC3B and p62 proteins were immunohistochemically evaluated in order to determine whether the injection of TTC influenced the regulation of autophagy in prion diseases. Immunohistochemical determination of these two autophagy markers revealed differential regulation of autophagic mechanisms in mice treated with TTC injection. Regarding LC3B, an increase of immunolabelling was noticed in all brain areas of both C57BL6 and Tg338 TTC-treated mice compared to their controls (Fig. 5B). This difference was statistically significant after performing the Mann–Whitney *U* test in mesencephalon, hypothalamus, thalamus and parietal cortex in C57BL6 mice and in all the evaluated brain areas except the hippocampus in Tg338 mice. LC3B immunostaining pattern was defined by consistent intraneuronal labeling affecting most of the neurons and by diffuse staining of the neuropil. Although they showed the same pattern, the control groups displayed a lower number of stained cells along with lower immunostaining intensities (Fig. 5A).

**Fig. 6:**
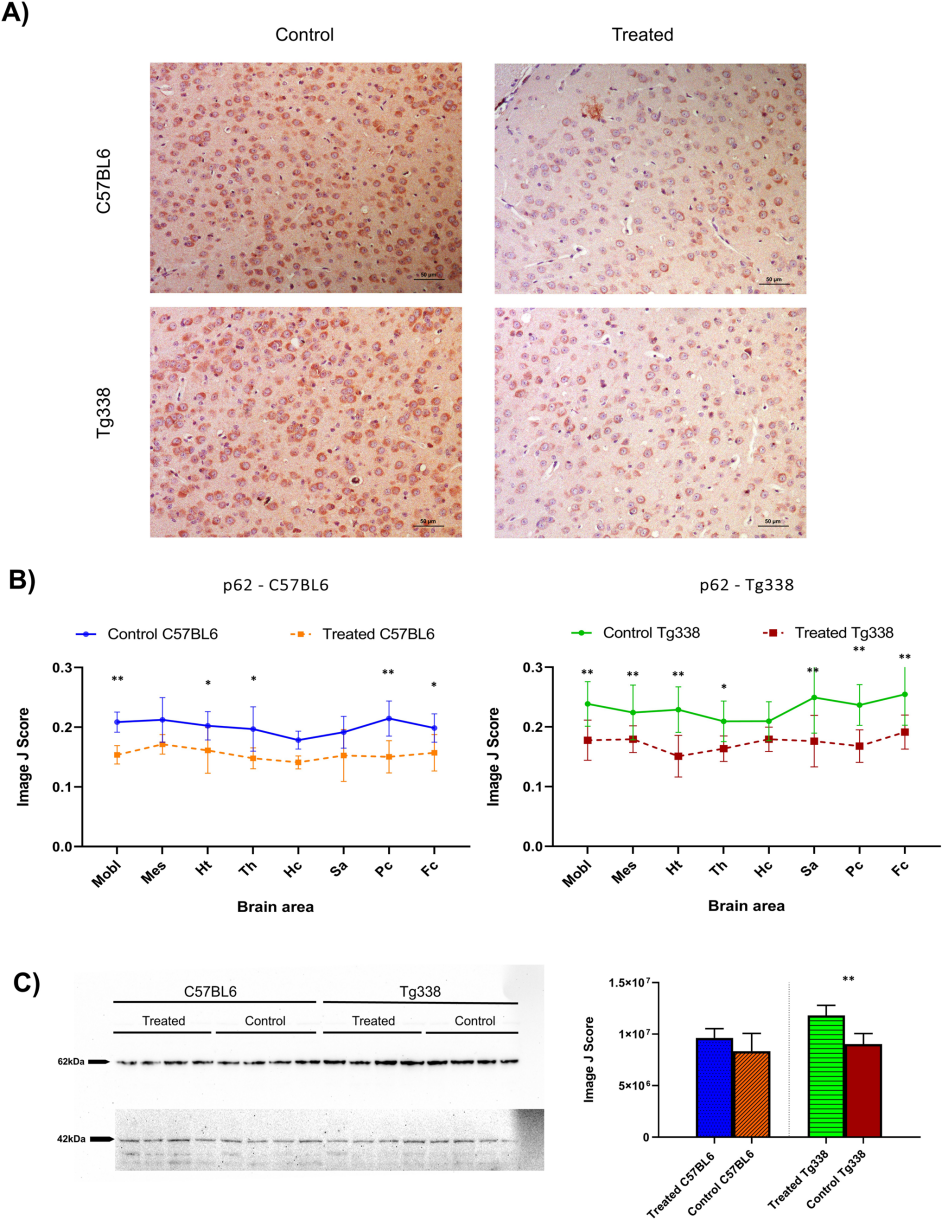
Assessment of p62 expression in Tg338 and C57BL6 prion infected mice after TTC intramuscular injection**. A** Representative images of p62 immunostaining in cerebral cortex of mice, where a cytoplasmic labeling can be observed. **B** Evaluation of p62 staining in brains of control and treated C57BL6 and Tg338 mice using the Image J software in the aforementioned brain areas. Treated mice of both lines displayed lower scores for p62 immunolabeling. Evaluation of significant differences was performed using the Mann–Whitney *U* test (*p < 0.05 and **p < 0.01) and significant differences were found in Mobl, Ht, Th, Pc and Fc in C57BL6 mice and in Mobl, Mes, Ht, Th, Pc, Sa and Fc in Tg338 mice. **C** Western Blot for the p62 protein in the frontal cortex different groups of mice. A single band at ~ 62 kDa confirms the specificity of the antibody. β-Actin immunodetection is shown at ~ 42 kDa. Significant differences were found between treated and untreated mice in the Tg338 line when using the Student’s *t* test (**p < 0.01)

On the contrary, treated mice of both lines showed clearly lower accumulation of p62 in comparison to their respective controls. TTC-injected mice displayed lower immunostaining scores in all the CNS analysed areas in both mouse lines. Differences were significant in medulla oblongata, hypothalamus, thalamus, frontal cortex and parietal cortex in C57BL6 mice and, as in LC3B evaluation, all brain areas but the hippocampus in Tg338 mice (Fig. 6B). The immunolabeling for p62 was characterized by neuronal and glial intracellular staining and granular staining of the neuropil. All mice presented this pattern, but treated mice showed lesser staining intensities and a lower number of stained cells (Fig. 6A).

The specificity of the antibodies and protein expression of these two autophagy markers were also tested by Western Blot in the frontal cortex of both treated and untreated C57BL6 and Tg338 mice. LC3B immunoblot revealed two bands at ~ 18 kDa and ~ 15 kDa, corresponding to the lipidated and non-lipidated forms of this protein (Fig. 5C). After signal band quantification of the interest proteins and normalization of the results by β-actin quantification, significant differences were found in the Tg338 line, where treated mice displayed higher protein levels than the control group, in agreement with the results of the immunohistochemistry. Regarding p62 immunoblot analyses, a single band at ~ 62 kDa was detected (Fig. 6C), and significant differences were found for Tg338 mice, the control group showing higher expression levels of this protein than treated animals, also in accordance with the immunohistochemical analyses for this protein.

**Fig. 7:**
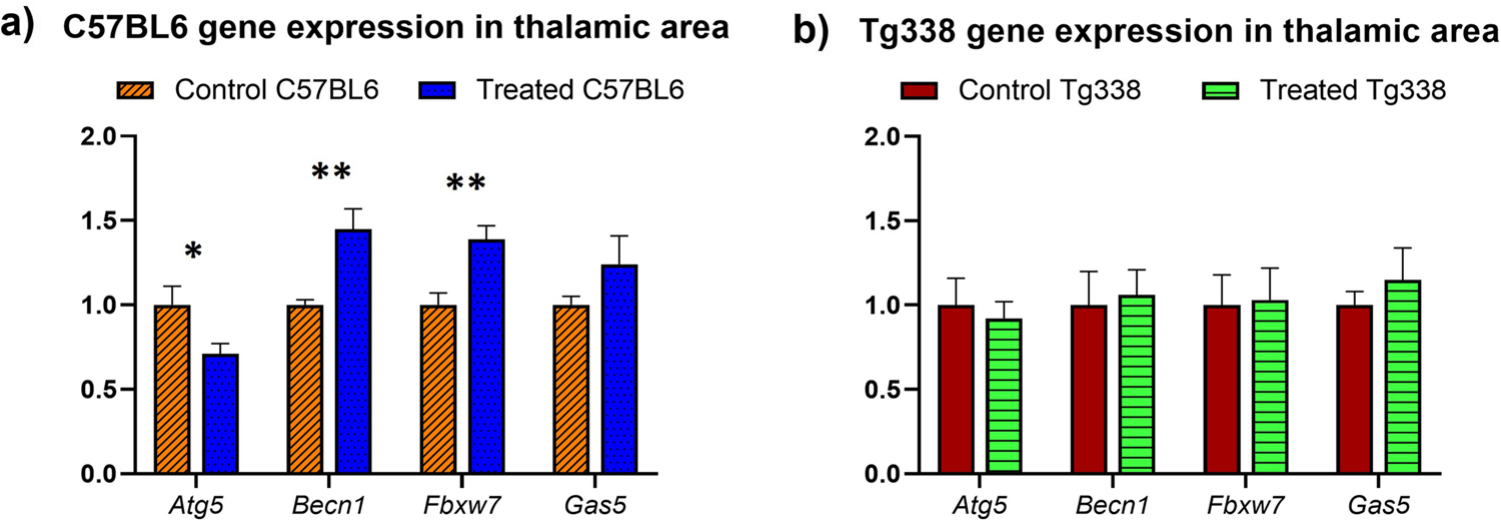
Autophagy-related gene expression profiles. mRNA expression profiles of *Atg5*, *Becn1*, *Fbxw7* and *Gas5* in the thalamus and hypothalamus of TTC-treated and controls C57BL6 and Tg338 mice. Relative expression levels are expressed as mean ± standard deviation. Normalization of results was performed using the expression of *Sdha* and *H6pd* housekeeping genes. The expression values were determined using the 2^−ddCt^ method and differences between experimental groups were analysed using the Student’s *t* test (*p < 0.05 and **p < 0.01)

Four markers of autophagy (*Atg5, Becn1, Fbxw7* and *Gas 5)* were analysed by real-time PCR in mice brain samples to determine their mRNA expression levels after TTC treatment. Expression of these genes was studied in the thalamic area (thalamus and hypothalamus) of Tg338 and C57BL6 TTC-treated and control mice. Figure 7 displays the mean dCt values of the analysed genes. *Becn1*, *Fbxw7* and *Gas5* displayed an upregulation and *Atg5* was downregulated in treated C57BL6 mice (Fig. 7A). Differences in gene expression between TTC-treated and control mice were more remarkable in C57BL6 mice, where *Atg5* displayed a significant decrease of the expression (*p* = 0.02), while *Becn1* and *Fbxw7* showed a significant upregulation (*p* = 0.0042 and *p* = 0.0043, respectively) in the treated group. Significant changes were not detected in Tg338 mice (Fig. 7B).

### Neuronal Survival Assessed Through NeuN Marker Expression Is Higher in Prion Diseased Mice Treated with TTC

**Fig. 8:**
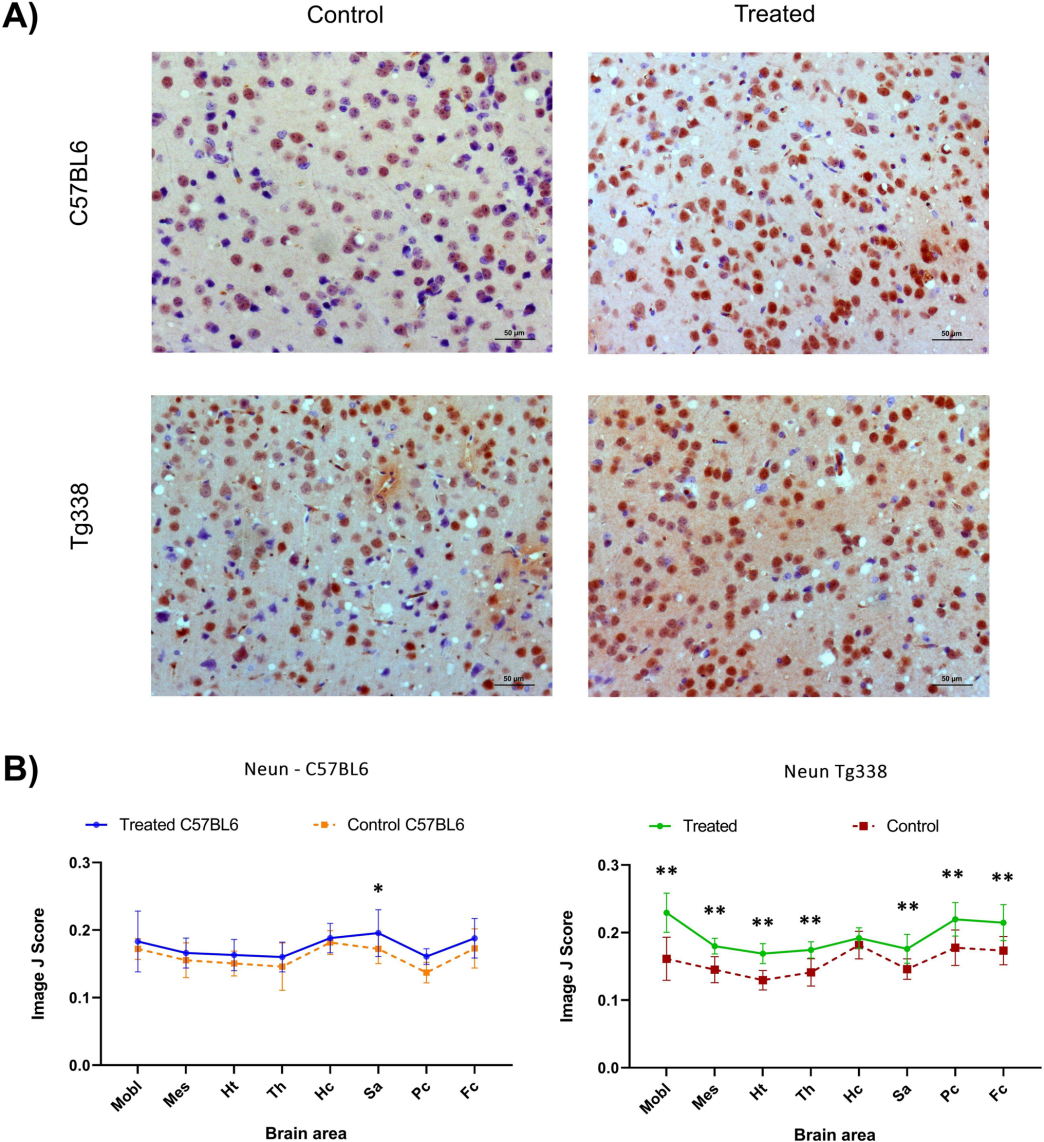
Immunohistochemical analyses of NeuN in the brains of TTC-injected and control Tg338 and C57BL6 mice. A Representative pictures of different staining levels of NeuN immunostaining in cerebral cortex of C57BL6 and Tg338 mice, showing a nuclear and perinuclear staining in neurons. **B** Comparison of the evaluation (using the Image J software) of the NeuN immunostaining in TTC-injected and control groups of Tg338 and C57BL6 lines in medulla oblongata (Mobl), mesencephalon (Mes), hypothalamus (Ht), thalamus (Th), hippocampus (Hc), septal area/striatum (Sa), parietal cortex (Pc) and frontal cortex (Fc). A NeuN immunostaining increase was observed in all brain areas of treated mice, both in C57BL6 and Tg338 lines. Significant differences were evaluated using the Mann–Whitney *U* test (*p < 0.05 and **p < 0.01) and found in Sa in C57BL6 mice and in Mobl, Mes, Ht, Th, Pc, Sa and Fc in Tg338 mice

The NeuN protein is widely used to assess neuronal survival since it reflects the functional state of these cells. NeuN immunolabeling was observed as a strong immunolabeling of the nucleus along with a perinuclear staining affecting numerous cells (Fig. 8A). Treated mice from both lines showed increased neuronal survival in all the analysed brain areas since the control group of both lines displayed a lower number of immunolabeled neurons (Fig. 8). In C57BL6 mice, significant differences were found using the Mann–Whitney *U* test between the treated and control group in the septal area/striatum, while in Tg338 mice significant differences in NeuN expression appeared in all the analysed brain areas but the hippocampus (Fig. 8B).

## Discussion

Prion diseases are fatal neurodegenerative disorders in which the main pathogenic event is the conversion of PrP^C^ into PrP^Sc^, leading to the accumulation of PrP^Sc^ in the central nervous system and therefore causing neuronal dysfunction and death resulting in spongiform degeneration. The neuroprotective role of the non-toxic C-terminal fragment of tetanus toxin has been related to antiapoptotic and pro-survival pathways, resembling neurotrophins, and demonstrated in vitro [32, 44] and in vivo in murine models of ALS and SMA [30, 34, 35]. We report here the first assay with TTC in a model of prion-related neurodegeneration.

The role of apoptosis in prion-associated neurodegeneration is still unclear. This mechanism has been described in scrapie-infected mice several times [16, 17, 45, 46], and the activation of caspases and some alterations in apoptosis-related genes have been detected in the latest stages of human and animal prion diseases [15, 20, 47]. Despite this, when challenging knock-out mice for the pro-apoptotic proteins caspase-12 or Bax with prions, no significant pathogenic changes or disease progression were found [48, 49]. Hence, it has been suggested that in these diseases, apoptosis may not be especially important for the onset and progression of the disease but a late process or, alternatively, that the main cell death mechanism in prion diseases might involve a non-apoptotic pathway [14].

TTC has been described as an anti-apoptotic molecule that binds to Trk receptors activating Trk-dependent signaling. This leads to the activation of pro-survival pathways such as MAPK/ERK and PI-3 K, which inhibit caspase-dependent apoptosis [31]. Reduction of apoptosis due to this molecule has already been described in ALS murine models, where decreased levels of the active form of caspase-3 after TTC injection have been reported [34]. In this study, we have observed that TTC also reduces caspase-3 related apoptosis in prion-infected mice. TTC treated-mice presented significantly lower levels of the active form of caspase-3 in their brains, thus confirming that, as in other neurodegenerative diseases, TTC could affect antiapoptotic pathways.

The cellular process of autophagy has several functions within we find response to nutrient starvation, intracellular clearance or elimination of pathogens [50], and it is also involved in the degradation of damaged proteins therefore being one of the most important protein homeostasis systems [5]. These functions make autophagy a pro-survival mechanism, but it has also been reported to mediate a non-apoptotic cellular death [51]. Due to this, the impairment of autophagy has been related to different pathologies, including neurodegenerative diseases [52]. Impaired protein homeostasis has been reported as one of the main causes of prion-associated toxicity [13], and autophagy has been claimed to have a neuroprotective role against prion-mediated neurotoxicity [13, 53, 54]. Despite its initial cytoprotective role, it has also been proposed that when autophagy progresses unrestrained over a certain threshold, it might lead to the upregulation of cell death markers and activation of apoptosis or other cell death modalities [55].

The analysis of LC3 (microtubule-associated protein 1 light chain 3, which presents three isoforms — LC3A, LC3B and LC3C — and is present in the autophagosome membrane [56]) and p62 (also known as SQSTM1, sequestosome 1, which acts as a substrate in autophagic degradation [57]) together is a good approach for the evaluation of autophagic activity [58]. LC3B is one of the most used markers to monitor autophagy in mammals [59], and p62 accumulation reveals a dysregulation of autophagic mechanisms [60]. Moreover, the downregulation of LC3B and the accumulation of p62 have been reported in prion-infected clinical mice compared to non-diseased mice, suggesting an impairment of autophagy at the late stage of prion infection [12]. In our study, after TTC-injection, scrapie-diseased mice presented differences in the regulation of autophagy proteins compared to their controls. While LC3B showed an increase of immunolabelling in TTC-treated mice, p62 immunostaining intensity was lower in this group compared to their controls, which reflects an induction of autophagy as a result of this treatment. These differences were present in all the analysed brain areas, and in both Tg338 and C57BL6 mice.

To complement the autophagic-related protein expression studies, we assessed the autophagy at a gene-expression level through the analysis of the markers of autophagy *Becn1*, *Fbxw7*, *Gas5* and *Atg5* [36]. After injection of TTC, we observed an upregulation of *Becn1*, *Fbxw7* and *Gas5* and a downregulation of *Atg5* transcripts. Downregulation of these positive autophagy gene regulators (*Becn1*, *Fbxw7*, *Gas5* and *Atg5*) has been described in the CNS of scrapie-infected mice (both wild-type or transgenic) and naturally scrapie infected sheep [12, 61]. Therefore, the observed upregulation of *Becn1*, *Fbxw7* and *Gas5* could reflect a recovery of autophagy dysregulation induced by prions after TTC injection. Gene expression differences were more noticeable in C57BL6 mice than in Tg338 mice. PrP^C^ seems to play a role in the regulation of the autophagic process [62], and then overexpression of ovine PrP^C^ in Tg338 could affect autophagy modulation, at least at the transcript level as p62 and LC3B levels were similar in both models. Differences in autophagy-related proteins and gene expression under TTC treatment show an effect of TTC in the autophagic machinery regulation involved in prion diseases. Similarly, TTC alters/modulates autophagy mechanisms in muscle and spinal cord of murine SMA models [35] where TTC induces downregulation of p62 and *Atg5* as in our assay but, in contrast to our results, decreased LC3 and *Becn1* levels. As mentioned above, the genetic background can be crucial in the response of animals to TTC treatment, mainly at the transcript level. This or the moment and tissue used for the analysis could explain these differences.

The NeuN protein, localized in the nucleus and perinuclear cytoplasm of most neurons in the central nervous system of mammals, reflects the functional state of these cells [63] and therefore is used as a marker of neuronal survival. This neuronal marker has been reported to decrease in prion infected mice during prion disease due to severe neuronal loss [64]. Our results show that NeuN immunolabelling was higher in scrapie diseased mice after TTC-injection in both mouse lines. Although sometimes mild, this increase on neuronal survival could be observed in all brain areas. The increase in neuronal survival could be observed in the treated group of both lines, but the statistical significance was higher in Tg338. The overexpression of PrP^C^ is known to affect several factors, from different molecular pathways [65] to strain selection in prion diseases [66]. Moreover, PrP^C^ has been reported to display a neuroprotective role [67]. Therefore, Tg338 PrP^C^ overexpression could be affecting not only autophagy mechanisms, as previously proposed, but also the response of other survival pathways to TTC administration. The fact that neuronal survival was higher in the TTC-treated group even though spongiosis was similar in TTC-treated and control mice could be related to the reduction of the apoptosis observed in treated mice. 

The finding that TTC affected apoptosis and autophagy and that this was linked to an increase on neuronal survival in TTC-treated mice suggests that TTC is able to reduce apoptosis and to regulate autophagy. Moreover, the differences in p62, LC3 and NeuN expression between control and TTC-injected groups could be observed in all brain areas, suggesting that TTC is able to diffuse to the whole brain.

Currently, there are no effective therapeutic or prophylactic treatments for prion diseases. Given the complex pathogenesis of these disorders, a wide variety of therapeutic strategies has been proposed, those aiming at reducing PrP^Sc^ accumulation being of special importance, since this protein is the underlying neurotoxic agent [13]. There is experimental evidence that the induction of autophagic activity through drugs reduces the levels of PrP^Sc^ [6, 68], but it has been observed that the administration of these drugs once neuroinvasion has already taken place does not cause a clear degradation of PrP^Sc^ deposits in the CNS. Thus, it is plausible that autophagic regulation after intracerebral prion inoculation does not lead to a decrease in PrP^Sc^ deposition. In agreement with this, we observed that, although TTC affects autophagy, it does not avoid PrP^Sc^ accumulation. This explains the fact that both TTC-treated and control mice developed similar PrP^Sc^ and spongiosis scores, and as a result their survival periods were similar. However, given the autophagy regulatory characteristics that TTC shows in prion disease models, further investigations on TTC effects after prion oral or intraperitoneal inoculation or in prion-infected cultured cells could be useful to elucidate the effects of TTC on autophagy in these diseases, and its connection with PrP^Sc^ propagation.

In summary, even though TTC has not affected vacuolation or PrP^Sc^ accumulation, it shows neuroprotective properties through the regulation of autophagic mechanisms and reduction of caspase-dependent apoptosis, thus leading to an increase of neuronal survival. These preliminary findings reinforce the role of TTC as a regulator of apoptosis and autophagy, and more importantly, as a neuroprotective molecule.

## Data Availability

The data presented in this study are available within the article text, figures and supplementary materials.

## Declarations

### Disclaimer

The funders had no role in study design, data collection and analysis, decision to publish, or preparation of the manuscript.

## References

[1] Prusiner S (1982). Novel proteinaceous infectious particles cause scrapie. Science.

[2] Pattison IH, Jones KM (1967). The possible nature of the transmissible agent of scrapie. Vet Rec.

[3] Frost B, Diamond MI (2010). Prion-like mechanisms in neurodegenerative diseases. Nat Rev Neurosci.

[4] Liberski PP, Sikorska B, Bratosiewicz-Wasik J, Carleton Gajdusek D, Brown P (2004). Neuronal cell death in transmissible spongiform encephalopathies (prion diseases) revisited: from apoptosis to autophagy. Int J Biochem Cell Biol.

[5] Rubinsztein DC (2006). The roles of intracellular protein-degradation pathways in neurodegeneration. Nature.

[6] Heiseke A, Aguib Y, Schatzl HM (2010). Autophagy, prion infection and their mutual interactions. Curr Issues Mol Biol.

[7] Nassif M, Hetz C (2014). Targeting autophagy in ALS: a complex mission. Autophagy.

[8] Boellaard JW, Schlote W, Tateishi J (1989). Neuronal autophagy in experimental Creutzfeldt-Jakob’s disease. Acta Neuropathol.

[9] Xu Y, Tian C, Wang S-B, Xie W-L, Guo Y, Zhang J, Shi Q, Chen C (2014). Activation of the macroautophagic system in scrapie-infected experimental animals and human genetic prion diseases. Autophagy.

[10] Moon J-H, Lee J-H, Nazim U Md., Lee Y-J, Seol J-W, Eo S-K, Lee J-H, Park S-Y (2016). Human prion protein-induced autophagy flux governs neuron cell damage in primary neuron cells. Oncotarget.

[11] Llorens F, Thüne K, Sikorska B, Schmitz M, Tahir W, Fernández-Borges N, Cramm M, Gotzmann N et al (2017) Altered Ca2+ homeostasis induces calpain-cathepsin axis activation in sporadic Creutzfeldt-Jakob disease. Acta Neuropathol Commun 5. 10.1186/s40478-017-0431-y 10.1186/s40478-017-0431-y PMC5408381 28449707

[12] López-Pérez Ó, Toivonen JM, Otero A, Solanas L, Zaragoza P, Badiola JJ, Osta R, Bolea R (2019). Impairment of autophagy in scrapie-infected transgenic mice at the clinical stage. Lab Invest.

[13] Goold R, McKinnon C, Tabrizi SJ (2015). Prion degradation pathways: Potential for therapeutic intervention. Mol Cell Neurosci.

[14] Mays CE, Soto C (2016). The stress of prion disease. Brain Res.

[15] Serrano C, Lyahyai J, Bolea R, Varona L, Monleón E, Badiola JJ, Zaragoza P, Martín-Burriel I (2009) Distinct spatial activation of intrinsic and extrinsic apoptosis pathways in natural scrapie: association with prion-related lesions. Vet Res 40. 10.1051/vetres/2009024 10.1051/vetres/2009024 PMC2701179 19401142

[16] Sisó S, Puig B, Varea R, Vidal E, Acín C, Prinz M, Montrasio F, Badiola J (2002). Abnormal synaptic protein expression and cell death in murine scrapie. Acta Neuropathol.

[17] Williams A, Lucassen PJ, Ritchie D, Bruce M (1997). PrP Deposition, microglial activation, and neuronal apoptosis in murine scrapie. Exp Neurol.

[18] Gray F, Chrétien F, Adle-Biassette H, Dorandeu A, Ereau T, Delisle M-B, Kopp N, Ironside JW (1999). Neuronal apoptosis in Creutzfeldt-Jakob disease. J Neuropathol Exp Neurol.

[19] Lyahyai J, Bolea R, Serrano C, Vidal E, Pumarola M, Badiola JJ, Zaragoza P, Martín-Burriel I (2007). Differential expression and protein distribution of Bax in natural scrapie. Brain Res.

[20] Lyahyai J, Bolea R, Serrano C, Monleón E, Moreno C, Osta R, Zaragoza P, Badiola JJ (2006). Correlation between Bax overexpression and prion deposition in medulla oblongata from natural scrapie without evidence of apoptosis. Acta Neuropathol.

[21] Otero A, Betancor M, Eraña H, Fernández Borges N, Lucas JJ, Badiola JJ, Castilla J, Bolea R (2021) Prion-associated neurodegeneration causes both endoplasmic reticulum stress and proteasome impairment in a murine model of spontaneous disease. Int J Mol Sci 22. 10.3390/ijms22010465 10.3390/ijms22010465 PMC7796520 33466523

[22] Kristiansen M, Messenger MJ, Klöhn P-C, Brandner S, Wadsworth JDF, Collinge J, Tabrizi SJ (2005). Disease-related prion protein forms aggresomes in neuronal cells leading to caspase activation and apoptosis*. J Biol Chem.

[23] Farrar JJ, Yen LM, Cook T, Fairweather N, Binh N, Parry J, Parry CM (2000). Tetanus. J Neurol Neurosurg Psychiatry.

[24] Schiavo G, Matteoli M, Montecucco C (2000). Neurotoxins affecting neuroexocytosis. Physiol Rev.

[25] Herreros J, Lalli G, Schiavo G (2000). C-terminal half of tetanus toxin fragment C is sufficient for neuronal binding and interaction with a putative protein receptor. Biochem J.

[26] Herreros J, Lalli G, Montecucco C, Schiavo G (2000). Tetanus toxin fragment C binds to a protein present in neuronal cell lines and motoneurons. J Neurochem.

[27] Coen L, Osta R, Maury M, Brulet P (1997). Construction of hybrid proteins that migrate retrogradely and transynaptically into the central nervous system. Proc Natl Acad Sci.

[28] Chian R-J, Li J, Ay I, Celia SA, Kashi BB, Tamrazian E, Matthews JC, Bronson RT (2009). IGF-1: Tetanus toxin fragment C fusion protein improves delivery of IGF-1 to spinal cord but fails to prolong survival of ALS mice. Brain Res.

[29] Larsen KE, Benn SC, Ay I, Chian R-J, Celia SA, Remington MP, Bejarano M, Liu M (2006). A glial cell line-derived neurotrophic factor (GDNF):tetanus toxin fragment C protein conjugate improves delivery of GDNF to spinal cord motor neurons in mice. Brain Res.

[30] Ciriza J, García-Ojeda M, Martín-Burriel I, Agulhon C, Miana-Mena F, Muñoz M, Zaragoza P, Brûlet P et al (2008) Antiapoptotic activity maintenance of brain derived neurotrophic factor and the C fragment of the tetanus toxin genetic fusion protein. Open Life Sci 3. 10.2478/s11535-008-0011-z

[31] Toivonen JM, Olivan S, Osta R (2010). Tetanus toxin C-fragment: the courier and the cure?. Toxins (Basel).

[32] Gil C, Chaib-Oukadour I, Pelliccioni P, Aguilera J (2000). Activation of signal transduction pathways involving trkA, PLCgamma-1, PKC isoforms and ERK-1/2 by tetanus toxin. FEBS Lett.

[33] Mendieta L, Venegas B, Moreno N, Patricio A, Martinez I, Aguilera J, Limon ID (2009). The carboxyl-terminal domain of the heavy chain of tetanus toxin prevents dopaminergic degeneration and improves motor behavior in rats with striatal MPP(+)-lesions. Neurosci Res.

[34] Moreno-Igoa M, Calvo AC, Penas C, Manzano R, Oliván S, Muñoz MJ, Mancuso R, Zaragoza P (2009). Fragment C of tetanus toxin, more than a carrier. Novel perspectives in non-viral ALS gene therapy. J Mol Med.

[35] Olivan S, Calvo AC, Rando A, Herrando-Grabulosa M, Manzano R, Zaragoza P, Tizzano EF, Aquilera J et al (2016) Neuroprotective effect of non-viral gene therapy treatment based on tetanus toxin c-fragment in a severe mouse model of spinal muscular atrophy. Front Mol Neurosci 9. 10.3389/fnmol.2016.00076 10.3389/fnmol.2016.00076 PMC4995219 27605908

[36] López-Pérez Ó, Badiola JJ, Bolea R, Ferrer I, Llorens F, Martín-Burriel I (2020) An update on autophagy in prion diseases. Front Bioeng Biotechnol 8. 10.3389/fbioe.2020.00975 10.3389/fbioe.2020.00975 PMC7481332 32984276

[37] Olivan S, Calvo AC, Gasco S, Munoz MJ, Zaragoza P, Osta R (2015). Time-point dependent activation of autophagy and the UPS in SOD1G93A mice skeletal muscle. PLoS One.

[38] Laude H, Vilette D, Le Dur A, Archer F, Soulier S, Besnard N, Essalmani R, Vilotte J-L (2002). New in vivo and ex vivo models for the experimental study of sheep scrapie: development and perspectives. CR Biol.

[39] Moreno-Martinez L, de la Torre M, Munoz MJ, Zaragoza P, Aguilera J, Calvo AC, Osta R (2020). Neuroprotective fragment C of tetanus toxin modulates IL-6 in an ALS mouse model. Toxins.

[40] Schulz-Schaeffer WJ, Tschöke S, Kranefuss N, Dröse W, Hause-Reitner D, Giese A, Groschup MH, Kretzschmar HA (2000). The paraffin-embedded tissue blot detects PrPSc early in the incubation time in prion diseases. Am J Pathol.

[41] Fraser H, Dickinson AG (1968). The sequential development of the brain lesions of scrapie in three strains of mice. J Comp Pathol.

[42] Fraser H, Dickinson AG (1973). Scrapie in mice. J Comp Pathol.

[43] Mustafa H, El Awdan S, Hegazy G, Abdel Jaleel G (2015) Prophylactic role of coenzyme Q10 and Cynara scolymus L on doxorubicin-induced toxicity in rats: Biochemical and immunohistochemical study. Indian J Pharmacol 47. 10.4103/0253-7613.169588 10.4103/0253-7613.169588 PMC4689020 26729958

[44] Chaib-Oukadour I, Gil C, Aguilera J (2004). The C-terminal domain of the heavy chain of tetanus toxin rescues cerebellar granule neurones from apoptotic death: involvement of phosphatidylinositol 3-kinase and mitogen-activated protein kinase pathways. J Neurochem.

[45] Kretzschmar HA, Giese A, Brown DR, Herms J, Keller B, Schmidt B, Groschup M (1997). Cell death in prion disease. J Neural Transm Suppl.

[46] Giese A, Kretzschmar HA (2001). Prion-induced neuronal damage–the mechanisms of neuronal destruction in the subacute spongiform encephalopathies. Curr Top Microbiol Immunol.

[47] Hetz C (2003). Caspase-12 and endoplasmic reticulum stress mediate neurotoxicity of pathological prion protein. EMBO J.

[48] True-Krob HL, Chiesa R (2015) The elusive role of the prion protein and the mechanism of toxicity in prion disease. PLoS Pathog 11. 10.1371/journal.ppat.1004745 10.1371/journal.ppat.1004745 PMC4423772 25951168

[49] Steele AD, Hetz C, Yi CH, Jackson WS, Borkowski AW, Yuan J, Wollmann RH, Lindquist S (2014). Prion pathogenesis is independent of caspase-12. Prion.

[50] Mizushima N, Hara T (2006). Intracellular quality control by autophagy: how does autophagy prevent neurodegeneration?. Autophagy.

[51] Kroemer G, Jaattela M (2005). Lysosomes and autophagy in cell death control. Nat Rev Cancer.

[52] Larsen KE, Sulzer D (2002). Autophagy in neurons: a review. Histol Histopathol.

[53] Forloni G, Artuso V, Roiter I, Morbin M, Tagliavini F (2013). Therapy in prion diseases. Curr Top Med Chem.

[54] Aguzzi A, Lakkaraju AKK, Frontzek K (2018). Toward therapy of human prion diseases. Annu Rev Pharmacol Toxicol.

[55] Mariño G, Niso-Santano M, Baehrecke EH, Kroemer G (2014). Self-consumption: the interplay of autophagy and apoptosis. Nat Rev Mol Cell Biol.

[56] Mizushima N, Yoshimori T (2014). How to interpret LC3 immunoblotting. Autophagy.

[57] Myeku N, Figueiredo-Pereira ME (2011). Dynamics of the degradation of ubiquitinated proteins by proteasomes and autophagy. J Biol Chem.

[58] Niklaus M, Adams O, Berezowska S, Zlobec I, Graber F, Slotta-Huspenina J, Nitsche U, Rosenberg R (2017). Expression analysis of LC3B and p62 indicates intact activated autophagy is associated with an unfavorable prognosis in colon cancer. Oncotarget.

[59] Suzuki H, Tabata K, Morita E, Kawasaki M, Kato R, Dobson RC, Yoshimori T, Wakatsuki S (2014). Structural basis of the autophagy-related LC3/Atg13 LIR complex: recognition and interaction mechanism. Structure.

[60] Tanida I (2011). Autophagosome formation and molecular mechanism of autophagy. Antioxid Redox Signal.

[61] López-Pérez Ó, Otero A, Filali H, Sanz-Rubio D, Toivonen JM, Zaragoza P, Badiola JJ, Bolea R et al (2019) Dysregulation of autophagy in the central nervous system of sheep naturally infected with classical scrapie. Sci Rep 9. 10.1038/s41598-019-38500-2 10.1038/s41598-019-38500-2 PMC6374525 30760781

[62] Shin H-Y, Oh J-M, Kim Y-S (2013). The functional role of prion protein (PrPC) on autophagy. Pathogens.

[63] Gusel’nikova VV, Korzhevskiy DE (2015). NeuN As a neuronal nuclear antigen and neuron differentiation marker. Acta Naturae.

[64] Jalland CMO, Scheffler K, Benestad SL, Moldal T, Ersdal C, Gunnes G, Suganthan R, Bjørås M et al (2016) Neil3 induced neurogenesis protects against prion disease during the clinical phase. Sci Rep 6. 10.1038/srep37844 10.1038/srep37844 PMC5122945 27886261

[65] Rachidi W, Vilette D, Guiraud P, Arlotto M, Riondel J, Laude H, Lehmann S, Favier A (2003). Expression of prion protein increases cellular copper binding and antioxidant enzyme activities but not copper delivery. J Biol Chem.

[66] Le Dur A, Lai TL, Stinnakre MG, Laisne A, Chenais N, Rakotobe S, Passet B, Reine F (2017). Divergent prion strain evolution driven by PrP(C) expression level in transgenic mice. Nat Commun.

[67] Lo RY-Y, Shyu W-C, Lin S-Z, Wang H-J, Chen S-S, Li H (2007). New molecular insights into cellular survival and stress responses: neuroprotective role of cellular prion protein (PrPC). Mol Neurobiol.

[68] Yao H, Zhao D, Khan SH, Yang L (2013). Role of autophagy in prion protein-induced neurodegenerative diseases. Acta Biochim Biophys Sin.

